# Investigating the impact of aspartame on Alzheimer’s disease through network toxicology and molecular docking

**DOI:** 10.3389/fnut.2025.1733469

**Published:** 2026-01-09

**Authors:** Lili Ge, Haitao Sun, Jianxin Zhang, Linlin Xu, Lei Ma, Zhi Jin

**Affiliations:** 1 Department of Traditional Chinese Medicine, The Second Qilu Hospital of Shandong University, Jinan, China; 2 Department of Neurology, The Second Qilu Hospital of Shandong University, Jinan, China; 3 Department of Pharmacy, Shanghe County People's Hospital, Jinan, China

**Keywords:** Alzheimer’s disease, aspartame, molecular docking, network toxicology, neurotoxicity

## Abstract

**Introduction:**

Alzheimer’s disease (AD) is a prevalent neurodegenerative disorder, and the relationship between its pathogenesis and environmental factors has garnered increasing scholarly interest. Aspartame, a widely utilized artificial sweetener, has potential neurotoxic effects that remain incompletely understood. This study employs network toxicology and molecular docking to speculate on the potential molecular mechanisms by which aspartame is involved in the pathological process of AD.

**Methods:**

By integrating data from multiple databases, including ChEMBL, SwissTargetPrediction, OMIM, and GeneCards, we obtained the shared targets of aspartame and AD. A protein–protein interaction (PPI) network was constructed using the STRING database and Cytoscape software to discern the core targets. Subsequently, Gene Ontology (GO) and Kyoto Encyclopedia of Genes and Genomes (KEGG) enrichment analyses were performed via the DAVID database, and molecular docking validation of the core targets was conducted using AutoDock Vina.

**Results:**

In this study, a total of 298 targets associated with aspartame and 2,042 targets related to AD were identified. Seventy-five common targets were discovered, with BCL2, PPARG, TNF, IL1β, MAPK3, ESR1, and CASP3 were hypothesized as key core targets. GO functional analysis indicated that these targets are predominantly involved in biological processes such as protein metabolism, neuroinflammation, apoptosis, and oxidative stress. Furthermore, KEGG pathway analysis revealed significant enrichment in pathways TNF signaling, MAPK signaling, and PI3K-Akt signaling, among others. Molecular docking studies have shown that aspartame has A certain binding affinity with some core targets.

**Discussion:**

It is speculated that aspartame may be involved in the key pathological processes of AD through multi-target and multi-pathway mechanisms, including neuroinflammation, apoptosis and amyloid-beta (Aβ) metabolism. This computational study speculates that aspartame, as an environmental exposure factor, is involved in the potential molecular mechanism of AD pathogenesis, thereby providing a theoretical basis for evaluating its neurotoxicity. Further experimental studies are needed in the future to confirm its biological effects.

## 1 Introduction

Alzheimer’s disease (AD) is a progressive neurodegenerative disorder marked by cognitive decline and abnormal behavior (1–3). By 2024, an estimated 6.9 million Americans aged 65 and older will have Alzheimer’s dementia, potentially rising to 13.8 million by 2060 without medical advances (4). The aging population contributes to its increasing prevalence, posing a significant public health challenge (5, 6). AD pathogenesis involves multiple pathological processes, including amyloid-beta (Aβ) protein deposition, neurofibrillary tangles from Tau protein phosphorylation, chronic neuroinflammation, and disrupted insulin signaling in the brain, alongside genetic factors and environmental exposures (2, 3, 7–9). Recent research has increasingly indicated that the artificial sweetener aspartame, along with its metabolites, possesses the capability to traverse the blood–brain barrier, thereby inducing oxidative stress responses and enhancing neuroinflammation. This process may contribute to and expedite neuronal degeneration (10–12).

Aspartame, a prevalent artificial sweetener, is extensively utilized in a variety of low-calorie foods and beverages. Although its safety has been endorsed by certain regulatory bodies, a growing body of neurotoxicity research suggests that its metabolites, including phenylalanine, aspartic acid, and methanol, can permeate the blood–brain barrier, accumulate within the central nervous system, and potentially initiate pathological processes associated with the onset of AD (10, 13–15). Empirical evidence indicates that aspartame may exacerbate the aberrant deposition of Aβ protein and the hyperphosphorylation of Tau protein by promoting oxidative stress in the brain, compromising the integrity of neuronal cell membranes, and activating neuroinflammatory pathways (10, 14, 16). Despite the current insufficiency of epidemiological evidence and the ongoing controversy surrounding mechanistic research, numerous experiments indicate that prolonged and excessive consumption of aspartame may lead to neuronal dysfunction and cognitive impairment (13, 17, 18). Considering the widespread use of aspartame in food products and its potential biological effects on the central nervous system, it is of substantial neurotoxicological and public health importance to conduct comprehensive research into the molecular mechanisms and population risks associated with AD.

To investigate the potential link between aspartame and AD, this study employed a comprehensive computational approach that integrates network toxicology, and molecular docking to systematically examine the neurotoxic mechanisms involved. A multi-dimensional interaction network comprising “compound - target - pathway” was developed using network toxicology to identify AD-related signaling pathways and biological processes that may be influenced by aspartame. Molecular docking was utilized to simulate the binding modes and affinities of aspartame with key targets, thereby assessing its potential for interference. This integrated methodology facilitates a systematic elucidation of the multi-target mechanisms through which aspartame may contribute to the progression of AD, providing a theoretical foundation for environmental health risk assessments. Additionally, it offers a computational toxicology framework for evaluating the neurotoxicity of food additives.

## 2 Methods

### 2.1 Identification of toxicological targets for aspartame

The two-dimensional chemical structure of aspartame and its standard SMILES notation were retrieved from the PubChem database.^1^ This SMILES expression was subsequently submitted to the SwissTarget Prediction online platform,^2^ where the species was set to “*Homo sapiens*” to predict targets and the targets whose credibility value was 0 were removed (19). Concurrently, a search was conducted in the ChEMBL database^3^ using “Aspartame” as the keyword to identify experimentally verified human targets. By integrating and deduplicating the targets identified from these two platforms, a set of potential toxicological action targets for aspartame was obtained.

### 2.2 Acquisition and intersection analysis of AD

The search term “Alzheimer’s disease” was utilized to identify relevant targets from the OMIM database^4^ and the GeneCards database.^5^ In the GeneCards database, a correlation score threshold of greater than 20 was applied to filter for high-confidence targets (20). The search results from both databases were integrated, and redundant genes were eliminated to construct a comprehensive target set for AD. Subsequently, the Microscopic Letter online analysis platform^6^ was employed to map potential targets of aspartame against the AD target set. The intersection of these datasets was identified to determine common targets, and a Venn diagram was generated to visualize the overlap.

### 2.3 Construction of PPI networks and identification of core targets

To examine the interaction relationships among the intersecting targets, the list of common targets was imported into the STRING database (version 11.5), specifying “*Homo sapiens*” as the species. The minimum interaction confidence score was set to 0.40, and free nodes were excluded from the analysis. The resulting PPI network data were subsequently imported into Cytoscape software (version 3.9.1) for visualization and further analysis. The CytoHubba plugin was employed to apply five topological algorithms—Betweenness, Closeness, Degree, Maximum Clique Centrality (MCC), and Edge Percolated Component (EPC)—to take intersection eventually get the core target.

### 2.4 GO and KEGG enrichment analyses

To elucidate the primary targets involved in biological functions and associated signaling pathways, we employed the DAVID online bioinformatics resources for enrichment analysis.^7^ The analysis was conducted with the species parameter set to “*Homo sapiens*,” and a significance threshold was established at a *p*-value of less than 0.05. The analysis encompassed the three categories of GO —biological processes (BP), molecular functions (MF), and cellular components (CC)—as well as pathways from the KEGG. The enrichment results were ranked based on p-value and the number of genes included. Visualization was achieved through bar charts or bubble charts, which were created on the WeChat platform to identify the principal biological processes and signaling pathways through which aspartame influences AD.

### 2.5 Molecular docking

To assess the binding potential of aspartame to specific target proteins at the molecular level, molecular docking simulations were employed. The three-dimensional structure of aspartame was retrieved from the PubChem database and subsequently converted to mol2 format utilizing Open Babel software. The crystal structure of the target protein was acquired from the RCSB Protein Data Bank, with extraneous water molecules, primary ligands, and non-essential ions removed using PyMOL software. Preprocessing of both the receptor protein and the ligand molecule, including hydrogenation and the calculation of Gasteiger charges, was conducted using AutoDock Tools software, and the structures were saved in pdbqt format. Semi-flexible molecular docking was executed using AutoDock Vina, with the docking grid box’s center coordinates and dimensions defined according to the protein’s active sites. Upon completion of the docking process, the conformation exhibiting the lowest binding free energy was selected as the most stable binding mode, and the interactions were visually analyzed using PyMOL software.

## 3 Results

### 3.1 Identification of aspartame-induced targets in AD

**Figure 1:**
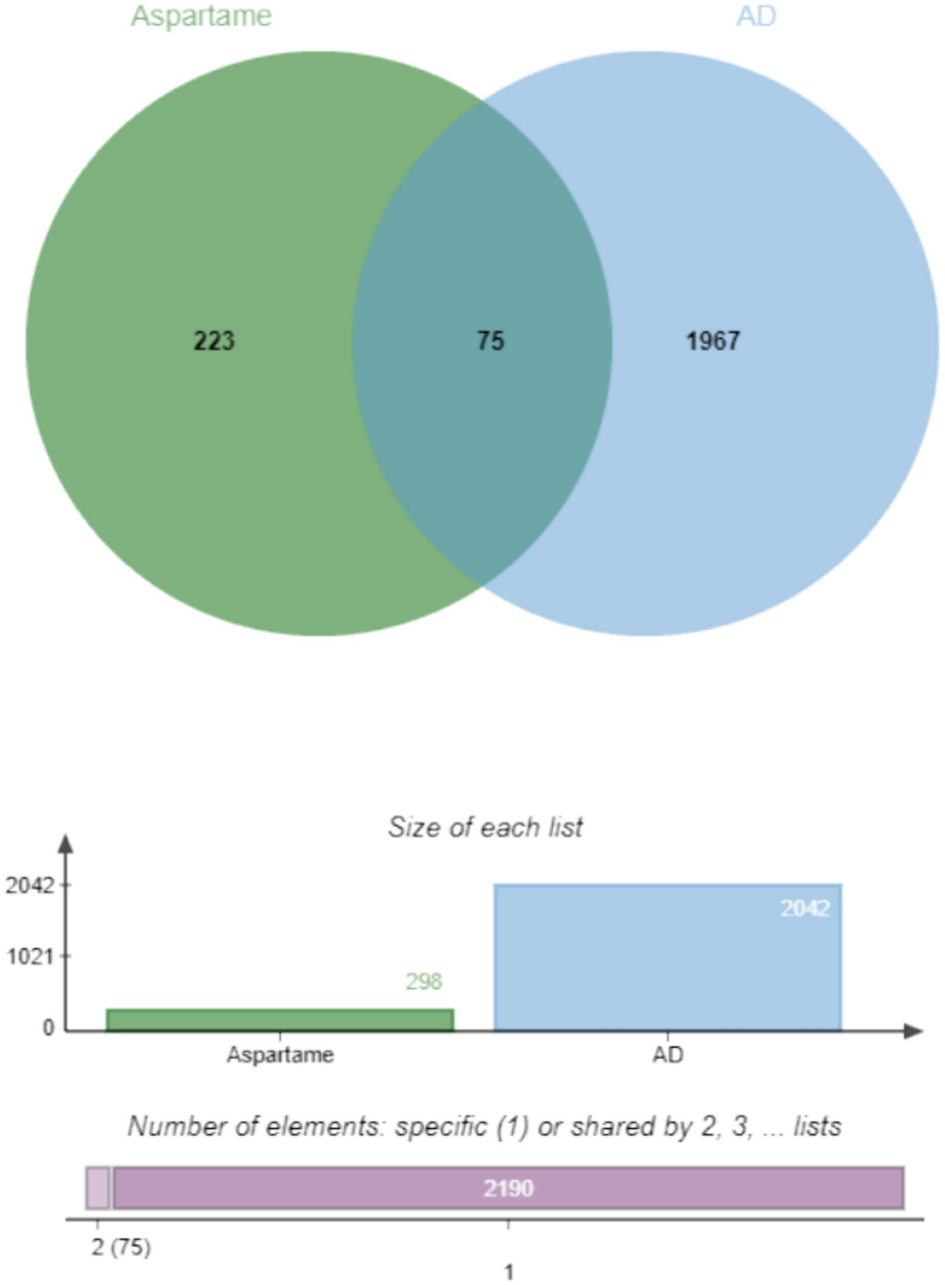
Venn diagrams of aspartame targets and Alzheimer’s disease targets.

To elucidate the relationship between aspartame and AD, this study initially conducted a systematic screening of their shared targets. By integrating predictive data from the ChEMBL and SwissTargetPrediction databases, 328 targets associated with aspartame were identified. Following the removal of duplicates, 298 potential action targets were confirmed. Concurrently, a search was performed using the GeneCards and OMIM databases with the keyword “Alzheimer’s disease.” After applying correlation score thresholds and eliminating redundancies, 2,042 AD-related targets were identified. By intersecting these two sets of targets, 75 common potential targets of aspartame and AD were ultimately identified (Figure 1). These targets may mediate the potential influence of aspartame on the pathological processes of AD.

### 3.2 Construction of PPI networks and identification of core targets

**Figure 2:**
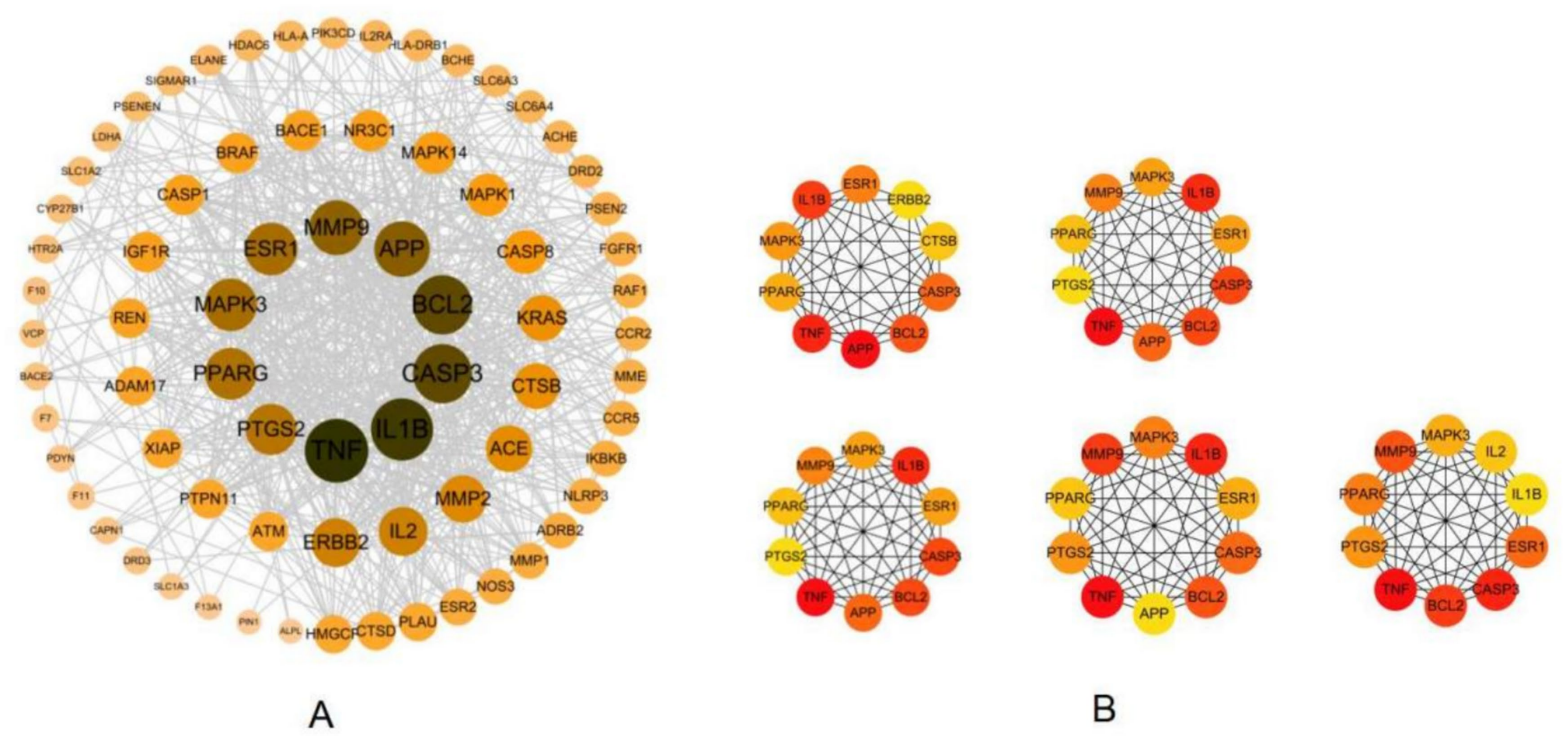
The PPI network of the potential targets and the core targets. **(A)** PPI network of the common targets. **(B)** Subnetwork of the top core targets identified.

To elucidate the interaction dynamics among the identified common targets, a total of 75 intersection targets were input into the STRING database to construct a PPI network. The species parameter was set to “*Homo sapiens*,” resulting in a network comprising 75 nodes (proteins) and 798 edges (interactions) (Figure 2A). To further pinpoint the hub proteins within this network, the PPI data were analyzed using Cytoscape software, employing the CytoHubba plugin. A comprehensive analysis was conducted using five topological algorithms: Betweenness, Closeness, Degree, MCC, and EPC. By focusing on targets that ranked highly across these algorithms, seven core targets were identified: BCL2, PPARG, TNF, IL1β, MAPK3, ESR1, and CASP3 (Figure 2B). These core targets are pivotal within the PPI network, indicating their potential significance in the pathological processes associated with aspartame-induced AD (Table 1).

**Table 1:** The PPI network of the potential targets and the core targets.

Algorithm	Name	Score	Degree
Betweenness	APP	670.365	43
	TNF	439.937	54
	IL1β	432.714	52
	BCL2	248.575	48
	CASP3	231.564	48
	ESR1	206.921	39
	MAPK3	159.839	39
	PPARG	159.194	38
	CTSB	129.355	30
	ERBB2	111.781	34
Closeness	TNF	63	54
	IL1β	62	52
	CASP3	60	48
	BCL2	60	48
	APP	57.5	43
	MMP9	57	42
	MAPK3	55.5	39
	ESR1	55.5	39
	PPARG	55	38
	PTGS2	54.5	37
Degree	TNF	54	54
	IL1β	52	52
	CASP3	48	48
	BCL2	48	48
	APP	43	43
	MMP9	42	42
	MAPK3	39	39
	ESR1	39	39
	PPARG	38	38
	PTGS2	37	37
MCC	TNF	6.32E+10	54
	CASP3	6.32E+10	48
	BCL2	6.32E+10	48
	MMP9	6.30E+10	42
	ESR1	6.22E+10	39
	PPARG	6.17E+10	38
	PTGS2	6.13E+10	37
	MAPK3	5.73E+10	39
	IL2	5.68E+10	34
	IL1β	4.86E+10	52
EPC	TNF	17.695	54
	IL1β	17.688	52
	CASP3	17.237	48
	BCL2	17.126	48
	MMP9	16.766	42
	MAPK3	15.965	39
	ESR1	15.932	39
	PPARG	15.705	38
	PTGS2	15.647	37
	APP	14.988	43

### 3.3 GO and KEGG enrichment analyses

**Figure 3:**
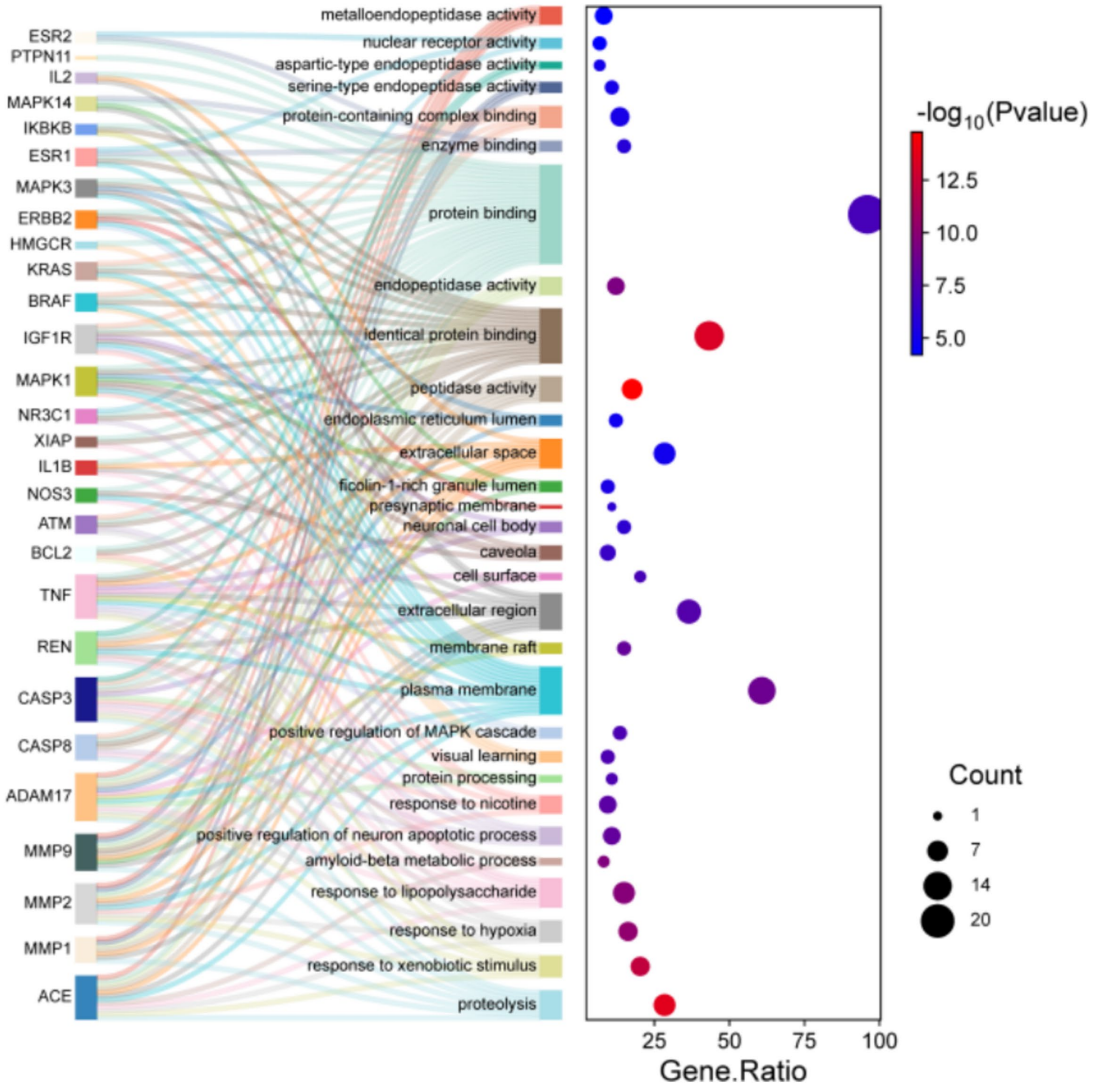
GO enrichment analysis of potential targets.

**Figure 4:**
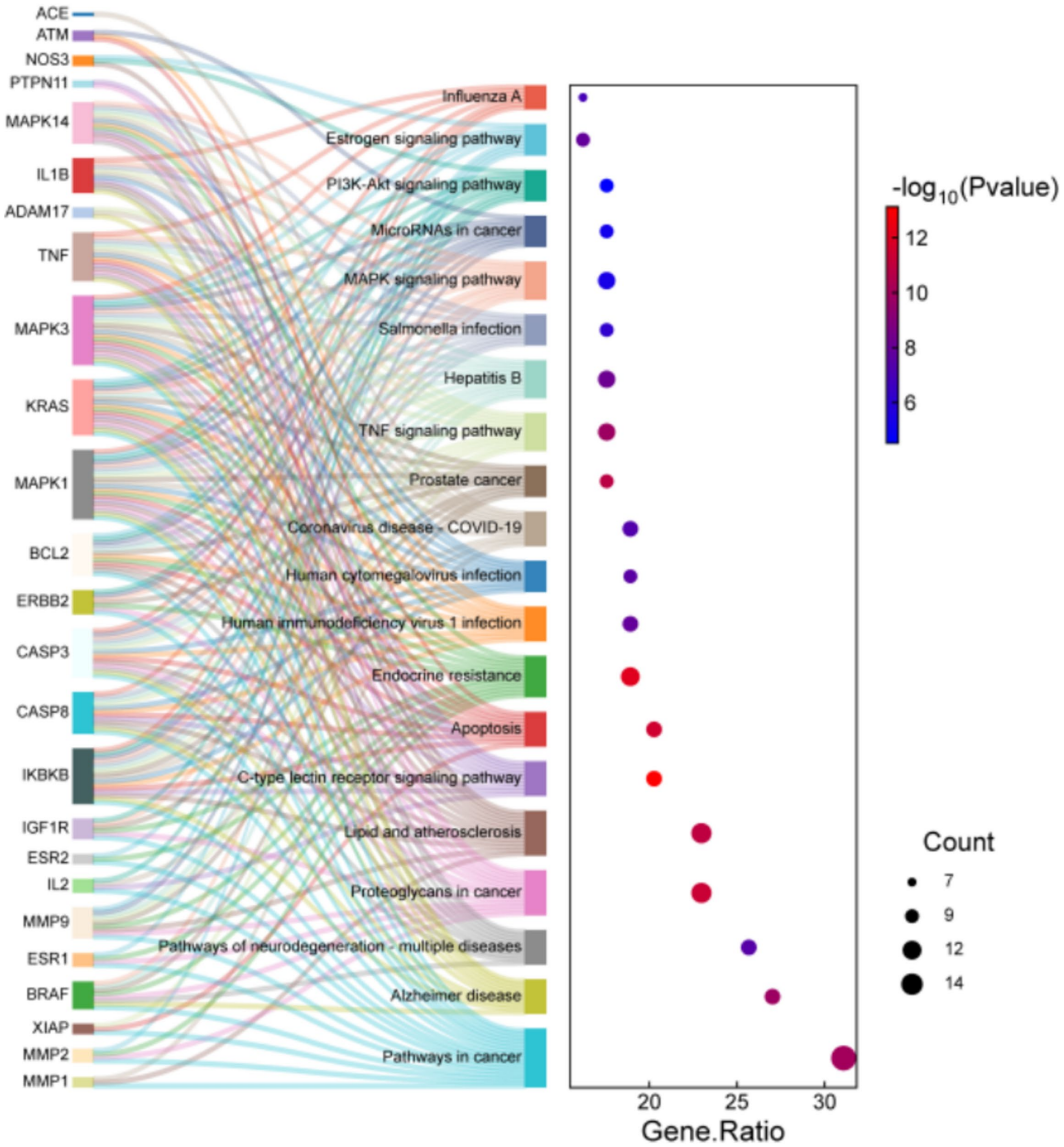
KEGG enrichment analysis of potential targets.

To systematically speculate the biological functions of the shared targets between aspartame and AD, this study employed GO analysis and KEGG pathway enrichment analysis. Seventy-five intersecting targets were input into the DAVID database, with the species parameter set to *Homo sapiens* and a significance threshold of *p* < 0.05. The analysis yielded 314 significantly enriched biological process, 54 cellular component, and 70 molecular function. Additionally, KEGG pathway analysis identified 148 significantly enriched signaling pathways. The results from the GO analysis highlighted several biological processes closely associated with AD pathology in which the intersecting targets are implicated. At the biological process level, targets were significantly enriched in key categories such as protein metabolism and homeostasis, encompassing processes like proteolysis, protein processing, and core pathological mechanisms of AD, including the Aβ metabolic process and the positive regulation of neuron apoptotic processes. Furthermore, cellular stress and inflammatory response pathways were also prominently represented. These findings from the KEGG enrichment analysis revealed that the intersecting target was significantly enriched in several critical signaling pathways. Notably, pathways directly associated with AD pathology include well-established pathways such as the TNF signaling pathway, MAPK signaling pathway, and PI3K-Akt signaling pathway. In summary, the enrichment analysis suggests that aspartame may potentially influence the onset and progression of AD by disrupting fundamental biological processes, including protein metabolism, inducing neuroinflammation and cellular stress, and facilitating neuronal apoptosis (Table 2, 3 and Figures 3, 4).

**Table 2:** GO enrichment analysis of potential targets.

Category	Term	Gene ratio	PValue	Count	Fold enrichment	FDR
BP	Proteolysis	28.38	2.77E-14	21	9.40	4.16E-11
BP	Response to xenobiotic stimulus	20.27	6.56E-13	15	15.31	4.94E-10
BP	Response to hypoxia	16.22	6.13E-11	12	17.69	3.08E-08
BP	Response to lipopolysaccharide	14.86	1.64E-10	11	19.90	6.18E-08
BP	Amyloid-beta metabolic process	8.11	2.97E-10	6	143.12	8.94E-08
BP	Positive regulation of neuron apoptotic process	10.81	5.16E-09	8	31.80	1.30E-06
BP	Response to nicotine	9.46	1.08E-08	7	43.73	2.32E-06
BP	Protein processing	10.81	3.64E-08	8	24.13	6.64E-06
BP	Visual learning	9.46	4.07E-08	7	35.32	6.64E-06
BP	Positive regulation of MAPK cascade	13.51	4.41E-08	10	13.59	6.64E-06
CC	Plasma membrane	60.81	1.82E-09	45	2.27	3.69E-07
CC	Membrane raft	14.86	4.04E-09	11	14.36	4.10E-07
CC	Extracellular region	36.49	1.99E-08	27	3.35	1.35E-06
CC	Cell surface	20.27	7.13E-08	15	6.30	3.62E-06
CC	Caveola	9.46	2.62E-07	7	26.07	1.06E-05
CC	Neuronal cell body	14.86	7.91E-07	11	8.17	2.67E-05
CC	Presynaptic membrane	10.81	9.20E-07	8	15.10	2.67E-05
CC	Ficolin-1-rich granule lumen	9.46	5.30E-06	7	15.64	1.35E-04
CC	Extracellular space	28.38	1.19E-05	21	2.95	2.68E-04
CC	Endoplasmic reticulum lumen	12.16	1.42E-05	9	8.06	2.87E-04
MF	Peptidase activity	17.57	1.67E-15	13	35.15	6.13E-13
MF	Identical protein binding	43.24	5.68E-14	32	4.67	1.04E-11
MF	Endopeptidase activity	12.16	4.04E-10	9	31.15	4.93E-08
MF	Protein binding	95.95	7.27E-08	71	1.35	6.65E-06
MF	Enzyme binding	14.86	1.45E-06	11	7.63	1.06E-04
MF	Protein-containing complex binding	13.51	6.64E-06	10	7.46	4.05E-04
MF	Serine-type endopeptidase activity	10.81	8.63E-06	8	10.76	4.51E-04
MF	Aspartic-type endopeptidase activity	6.76	1.03E-05	5	36.05	4.72E-04
MF	Nuclear receptor activity	6.76	5.26E-05	5	24.03	0.002138468
MF	Metalloendopeptidase activity	8.11	6.47E-05	6	14.03	0.002366904

**Table 3:** KEGG enrichment analysis of potential targets.

Term	Gene ratio	PValue	Count	Fold enrichment	FDR
Pathways in cancer	31.08	9.52E-11	23	5.24	9.52E-10
Alzheimer disease	27.03	1.56E-10	20	6.21	1.21E-09
Pathways of neurodegeneration - multiple diseases	25.68	3.49E-08	19	4.78	1.36E-07
Proteoglycans in cancer	22.97	4.55E-12	17	10.12	7.96E-11
Lipid and atherosclerosis	22.97	1.09E-11	17	9.56	1.53E-10
C-type lectin receptor signaling pathway	20.27	7.45E-14	15	17.35	5.21E-12
Apoptosis	20.27	2.85E-12	15	13.39	6.65E-11
Endocrine resistance	18.92	7.72E-13	14	17.17	2.70E-11
Human immunodeficiency virus 1 infection	18.92	1.25E-08	14	7.98	5.90E-08
Human cytomegalovirus infection	18.92	2.55E-08	14	7.52	1.05E-07
Coronavirus disease - COVID-19	18.92	4.72E-08	14	7.14	1.65E-07
Prostate cancer	17.57	1.49E-11	13	16.11	1.74E-10
TNF signaling pathway	17.57	1.52E-10	13	13.27	1.21E-09
Hepatitis B	17.57	5.89E-09	13	9.69	3.44E-08
Salmonella infection	17.57	6.94E-07	13	6.29	1.47E-06
MAPK signaling pathway	17.57	4.51E-06	13	5.26	7.17E-06
MicroRNAs in cancer	17.57	6.74E-06	13	5.06	9.63E-06
PI3K-Akt signaling pathway	17.57	3.00E-05	13	4.36	3.29E-05
Estrogen signaling pathway	16.22	1.26E-08	12	10.48	5.90E-08
Influenza A	16.22	1.23E-07	12	8.42	4.00E-07

### 3.4 Molecular docking

**Figure 5:**
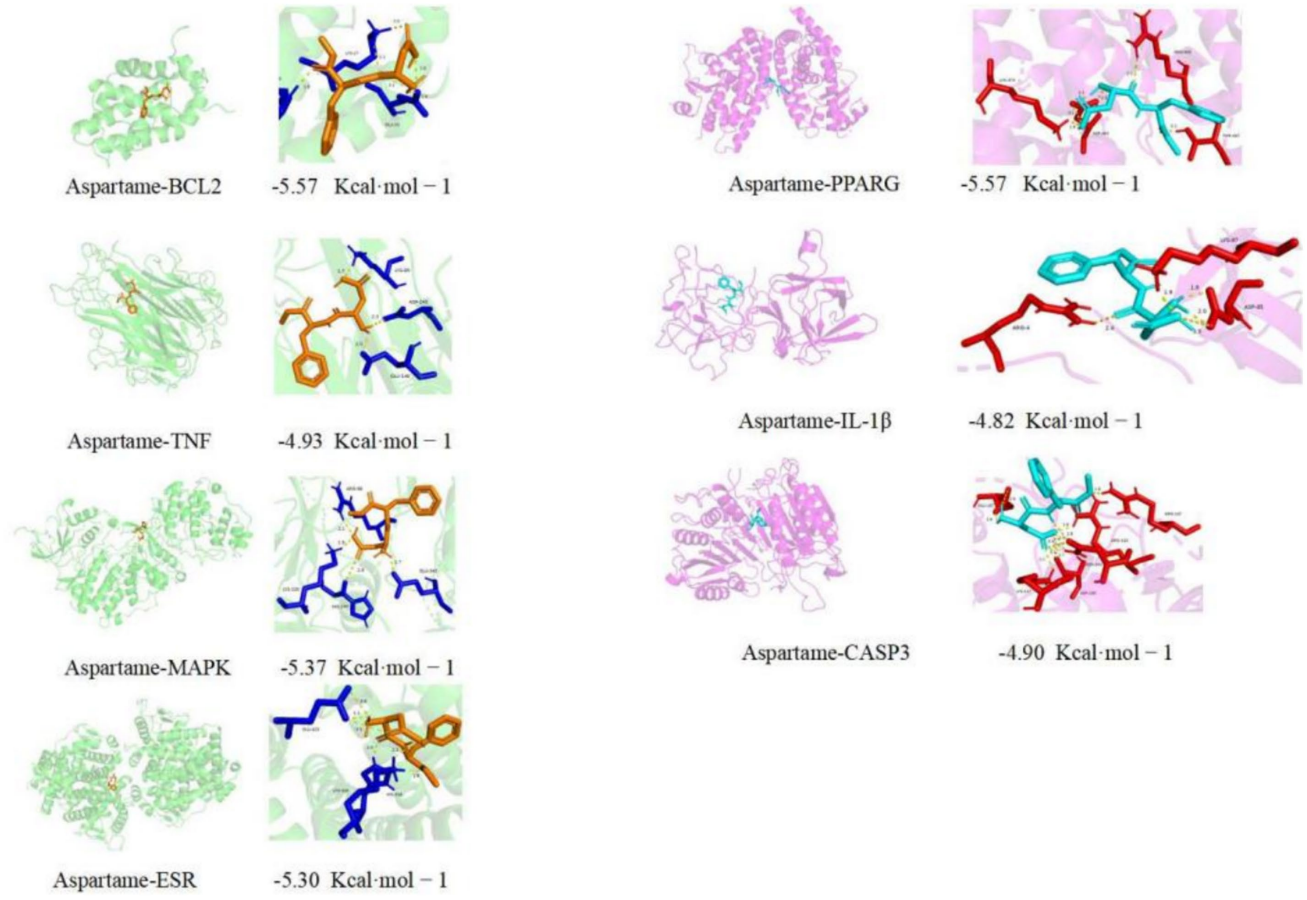
The results of molecular docking.

To assess the reliability of network pharmacological predictions at the structural level, this study utilized molecular docking to simulate the binding interactions and affinities between aspartame and seven core target proteins: BCL2, PPARG, TNF, IL1β, MAPK3, ESR1, and CASP3. Calculations were performed using AutoDock Vina software, with free energy (ΔG, in kcal·mol^−1^) serving as the primary metric for evaluating the stability of ligand-receptor binding. The molecular docking results demonstrated that aspartame exhibited good binding potential with all seven core targets (Table 4). This suggests that aspartame could theoretically form stable complexes with these target proteins. Further conformational analysis revealed that aspartame predominantly binds to the active sites of target proteins through intermolecular forces, including hydrogen bonds and hydrophobic interactions. The binding mode was elucidated using PyMOL software (Figure 5), which effectively illustrated the precise orientation of aspartame molecules within the protein binding cavity and highlighted the interactions with key amino acid residues. In summary, the molecular docking results, corroborated by computational simulation, confirmed that aspartame can stably bind to the core targets associated with AD. This finding provides structural biological support for the previously mentioned network pharmacological predictions and further reinforces the hypothesis that aspartame may play a role in the pathological process of AD through a multi-target mechanism of action.

**Table 4:** Binding capacity of aspartame to core targets.

Name	PDB ID	Binding capacity	Box coordinates			Box size		
			*x*	*y*	*z*	*x*	*y*	*z*
BCL2	2 mg5	−5.57	3.664	−4.153	−2.012	120	120	120
PPARG	2q59	−5.57	−20.142	0.947	−25.453	120	120	120
IL1β	4gai	−4.82	7.104	−16.433	−24.47	120	120	120
TNF	1a8m	−4.93	20.111	49.821	39.786	120	120	120
MAPK3	4qtb	−5.37	44.155	38.765	72.992	120	120	120
ESR1	3os8	−5.3	15.391	26.237	−45.217	120	120	120
BCL2	1g5m	−4.9	10,504	7.426	22.401	120	120	120

## 4 Discussion

Alzheimer’s disease, a progressive neurodegenerative disorder, is characterized by distinct pathological features, including the abnormal deposition of Aβ protein in the brain parenchyma, and the aggregation of hyperphosphorylated Tau protein within neurons (9, 21–23). Although the precise etiology of AD remains incompletely understood, it is widely accepted within the academic community that its pathogenesis arises from a complex interplay between genetic predisposition and prolonged environmental exposure (24–26). Beyond the classical Aβ cascade hypothesis and the Tau protein hypothesis, neuroinflammation has been identified as a critical factor driving the pathological progression of AD (27–30). Pro-inflammatory cytokines in the peripheral circulation can penetrate the central nervous system through alterations in blood–brain barrier permeability or by activating resident microglia in the brain, thereby initiating and sustaining a chronic inflammatory cascade within the cerebral environment (31–33). This persistent state of neuroinflammation not only directly contributes to neuronal damage but also exacerbates the aggregation of Aβ and the pathological alterations of Tau protein, thereby creating a vicious cycle that ultimately accelerates cognitive decline (34–36). Aspartame, a widely utilized artificial sweetener, is metabolized in the body into components such as phenylalanine, aspartic acid, and methanol. Existing research indicates that the neurotoxic effects induced by aspartame may involve multiple mechanisms, including neuroinflammation, oxidative stress, mitochondrial dysfunction, and apoptosis (10, 14, 16, 18, 37). In this context, a systematic investigation into the relationship between aspartame consumption and the onset and progression of AD, as well as its underlying mechanisms, is of substantial scientific and public health importance.

Utilizing network toxicology and molecular docking, this study peculated the potential molecular mechanisms through which aspartame may contribute to the pathogenesis of AD. By integrating and analyzing data from multiple databases, the study obtained 298 targets associated with aspartame and 2,042 targets related to AD. From this dataset, seven core targets—BCL2, PPARG, TNF, IL1β, MAPK3, ESR1, and CASP3— were speculated as potential mediators of the interaction between aspartame and AD. The molecular docking results indicated that aspartame had a good binding affinity with some core targets. It is speculated that aspartame may interfere with the signaling pathways related to AD through its interaction with these key proteins. Further KEGG enrichment analysis revealed significant enrichment in the TNF signaling pathway, MAPK signaling pathway, and PI3K-Akt signaling pathway. These pathways collectively regulate critical pathological processes in AD, such as neuroinflammatory response, apoptosis, and oxidative stress, thereby speculating on the potential mechanisms by which aspartame exposure may be involved in the occurrence and development of the disease.

This study hypothesizes that the primary targets identified are intricately linked to the key pathological mechanisms of AD. A central objective of AD research is to mitigate the accumulation of abnormal tau protein. Through the application of multispectral analysis on the brain tissues of individuals who succumbed to AD, it was observed that hyperphosphorylated tau protein was associated with numerous proteins from the BCL2 family. The findings of this research demonstrate that inhibiting BCL2 can substantially alleviate the tau protein burden (38). Additionally, the study reveals that aspartame can induce the upregulation of CASP3 expression and the downregulation of BCL2 expression (39). This theoretically represents one of the potential mechanisms that may increase the risk of AD. AD represents the most prevalent form of dementia, with neuroinflammation serving as a critical marker of its pathogenesis (35). TNF and its type 1 receptor are implicated not only in the neuroinflammatory processes associated with AD but also in amyloid protein formation through the regulation of *β*-secretase (40, 41). This dual role positions TNF as a promising candidate for therapeutic intervention in AD. Furthermore, TNF contributes to AD pathogenesis by mediating neuronal cell death. The inhibition of TNF-*α* holds potential as a therapeutic strategy to prevent AD and enhance cognitive function in populations at elevated risk for dementia (40, 41). TNF can also upregulate the expression of IL-1, thereby increasing the production of precursor substances essential for the formation of amyloid plaques, neurofibrillary tangles, and Lewy bodies (42). Additionally, TNF acts as a primary upstream mediator of inflammation within the choroid plexus tissue of AD patients (41). Previous research has demonstrated that aspartame can induce the formation of the pro-inflammatory factor TNF-α in serum (41, 43). Elevated levels of IL1β serve as pivotal mediators of inflammation and are expressed in the brain, particularly within the hippocampus, where they play an essential role in the regulation of memory and mood. Within the brain, IL1β is implicated in various processes, including neuronal proliferation, differentiation, apoptosis, and long-term potentiation (44). IL1β is also involved in the pathogenesis of AD and has an impact on cognitive function (44). Empirical studies have demonstrated that aspartame consumption can lead to an increase in the pro-inflammatory cytokine IL1β in the serum, thereby inducing systemic inflammation (30, 43). MAPK3 is involved in the regulation of cytokine, neurotransmitter, and hormone signal transduction, influencing gene expression, cell proliferation, and apoptosis. In the context of AD, the activation of the MAPK3 signaling pathway is closely associated with excessive microglial activation and the release of pro-inflammatory factors, which are critical pathological features of the disease (45). Inhibition of the MAPK3 pathway has been shown to reduce neuroinflammation and slow the progression of AD (46). Relevant research has demonstrated that MEP may activate the MAPK3 pathway, leading to an increased production and release of inflammatory mediators, thereby exacerbating neuronal damage and accelerating the pathological progression of AD. Aspartame has been shown to elevate levels of inflammatory mediators, potentially causing neuronal damage and further accelerating the pathological process of AD (10, 39). Apoptotic damage and subsequent cell death are critical factors in the neurodegeneration associated with AD. CASP3 plays a significant role in nervous system development and neuronal death under specific pathological conditions (47). Furthermore, both *in vitro* and *in vivo* studies have reported elevated expression and activation of CASP3 in AD models. Studies have confirmed that aspartame can induce the upregulation of CASP3 expression and promote cell apoptosis (47, 48). Additionally, Mendelian studies have identified that increased expression of the PPARG gene in the blood serves as an important protective factor against early-onset AD (49). ESR1 gene is ubiquitously expressed throughout the brain. Given the potential neuroprotective effects of estrogen, the ESR1 gene emerges as a candidate for modulating the development of AD (50–52). Notably, molecular docking studies have demonstrated that aspartame exhibits substantial binding affinity to these targets, indicating a possible direct interference with their physiological functions. Numerous experimental investigations have revealed that exposure to aspartame in animal models induces histopathological alterations across various brain regions, including increased oxidative stress markers and neuronal loss (10, 17). Furthermore, these studies have identified impairments in memory and learning, alongside behavioral and emotional dysfunctions in the animal models (13, 15, 17, 18). Abhilash M. et al. have highlighted that prolonged aspartame consumption can disrupt the antioxidant/pro-oxidant balance within the brain (14). The accumulation of neurotoxic metabolites in the central nervous system following extended dietary exposure raises concerns regarding potential damage to the cerebral cortex associated with aspartame. Aspartame has been shown to up-regulate CASP3 expression while down-regulating BCL2 expression, indicating the activation of apoptotic pathways and consequently exacerbating detrimental effects on the central nervous system (39). Furthermore, aspartame exposure is associated with elevated levels of pro-inflammatory cytokines, specifically TNF-*α* and IL1β, in the serum, which may precipitate systemic inflammation (43). Empirical evidence from animal studies has substantiated the impact of chronic aspartame exposure on oxidative stress within the cerebral tissue of albino rats (12). While molecular docking offers structural insights into target interactions, it is crucial to acknowledge the inherent limitations of computational simulations (53). Factors including the static nature of protein conformations, solvation effects, and *in vivo* metabolic kinetics can significantly influence the actual binding scenarios. Consequently, these findings primarily function as mechanistic hypotheses and require further validation of their biological relevance through *in vitro* experiments, such as those involving microglial and neuronal cell lines, as well as in vivo studies using animal models like APP/PS1 transgenic mice.

KEGG pathway enrichment analysis suggests that aspartame may play a role in the pathological processes of AD by modulating multiple signaling pathways. The TNF signaling pathway is implicated not only in the neuroinflammation associated with AD but also in amyloid protein formation through the regulation of *β*-secretase (40). Previous studies have demonstrated that aspartame can induce the production of the pro-inflammatory factor TNF-*α* in serum, thereby activating the TNF signaling pathway (43, 54). The MAPK signaling pathway has significant effects on memory, neuroinflammatory programming, and synaptic plasticity (45, 55). Consequently, clinical trials involving various MAPK inhibitors, which exhibit favorable safety profiles and minimal side effects, have yielded positive outcomes in AD patients, suggesting that targeting MAPK may effectively mitigate AD pathogenesis (45, 46, 56). Additionally, the PI3K/AKT signaling pathway has been identified as a pathway activated by multiple stimuli, including insulin, growth factors, cytokines, and cellular stress (48, 57–59). This pathway contributes to neuronal survival, regulates the phosphorylation of Tau protein, and facilitates the clearance of Aβ (60). The TNF signaling pathway and the MAPK signaling pathway collectively constitute the central network of the inflammatory response. Notably, TNF-*α* can further amplify the inflammatory signal by activating the MAPK cascade. It is important to highlight that the MAPK signaling pathway also plays a role in regulating the phosphorylation level of Tau protein (45). Dysregulation of these pathways results in the accumulation of Aβ, excessive phosphorylation of tau, and mitochondrial dysfunction, thereby forming a negative feedback loop that accelerates disease progression. Studies have indicated that aspartame may indirectly influence the formation of neurofibrillary tangles via this pathway. Research has demonstrated that aspartame can induce oxidative stress and activate these signaling pathways, modulating them through various mechanisms, including inflammation (TNFα, IL6, IL1β), enhanced oxidative stress (MDA), and the apoptotic pathway CASP3, ultimately leading to neuronal damage and promoting the onset and progression of AD (10).

As a computational exploratory study, the core value of this research lies in generating verifiable hypotheses. Although we have made every effort to correlate the prediction results with previous experimental evidence, the network pharmacology method itself has inherent limitations, such as the inability to simulate the complex pharmacokinetics, blood–brain barrier permeability and cell type specificity *in vivo*. The conclusion needs to be ultimately confirmed in subsequent experiments (53). Subsequent research should be further expanded in the following areas: Firstly, the regulatory effects of aspartame on the expression and function of core targets such as TNF and CASP3 should be verified using cell models, with particular emphasis on its impact within the microglia–neuron co-culture system. Secondly, transgenic animal models of AD should be employed to assess the in vivo effects of prolonged aspartame consumption on key pathological indicators, including Aβ deposition, Tau protein phosphorylation, and neuroinflammation. Finally, rigorous epidemiological studies should be designed to analyze the dose–response relationship between varying levels of aspartame intake and the risk of cognitive decline or AD onset. Establishing such a multi-tiered experimental validation framework is essential for a comprehensive evaluation of aspartame’s role as an environmental risk factor in the pathogenesis and progression of AD.

## 5 Conclusion

This study employed a combined approach of network toxicology and molecular docking to speculate on the potential role of aspartame in the pathogenesis of AD. The research results speculate that aspartame may be involved in this pathological process through a series of biological mechanisms, including interfering with neuroinflammatory responses, apoptosis and oxidative stress. Current research merely provides speculative evidence for the potential neurotoxicity of aspartame, and further empirical verification of its biological functions is still needed. The subsequent research should integrate *in vitro* neuronal models, transgenic animal experiments and population-based epidemiological studies to speculate on the possible mechanisms and clinical significance of aspartame in the occurrence and development of AD from multiple perspectives.

## Data Availability

The datasets presented in this study can be found in online repositories. The names of the repository/repositories and accession number(s) can be found in the article/supplementary material.

## Footnotes

1
https://pubchem.ncbi.nlm.nih.gov/

2
http://www.swisstargetprediction.ch/

3
https://www.ebi.ac.uk/chembl/

4
https://omim.org/

5
https://www.genecards.org/

6
http://www.bioinformatics.com.cn/

7 https://davidbioinformatics.nih.gov/, version 6.8
